# Finding of the optimal preparation and timing of endometrium in frozen-thawed embryo transfer: a literature review of clinical evidence

**DOI:** 10.3389/fendo.2023.1250847

**Published:** 2023-08-29

**Authors:** Ya-Wen Hsueh, Chien-Chu Huang, Shuo-Wen Hung, Chia-Wei Chang, Hsi-Chen Hsu, Tung-Chuan Yang, Wu-Chou Lin, Shan-Yu Su, Hsun-Ming Chang

**Affiliations:** ^1^ Department of Obstetrics and Gynecology, China Medical University Hospital, Taichung, Taiwan; ^2^ Department of Chinese Medicine, China Medical University Hospital, Taichung, Taiwan

**Keywords:** in vitro fertilization, frozen embryo transfer, endometrial receptivity, artificial cycle, natural cycle, true natural cycle, modified natural cycle, and mild stimulation cycle

## Abstract

Frozen-thawed embryo transfer (FET) has been a viable alternative to fresh embryo transfer in recent years because of the improvement in vitrification methods. Laboratory-based studies indicate that complex molecular and morphological changes in endometrium during the window of implantation after exogenous hormones with controlled ovarian stimulation may alter the interaction between the embryo and endometrium, leading to a decreased implantation potential. Based on the results obtained from randomized controlled studies, increased pregnancy rates and better perinatal outcomes have been reported following FET. Compared to fresh embryo transfer, fewer preterm deliveries, and reduced incidence of ovarian hyperstimulation syndrome were found after FETs, yet there is a trend of increased pregnancy-related hypertensive diseases in women receiving FET. Despite the increased application of FET, the search for the most optimal priming protocol for the endometrium is still undergoing. Three available FET protocols have been proposed to prepare the endometrium: i) natural cycle (true natural cycle and modified natural cycle) ii) artificial cycle (AC) or hormone replacement treatment cycle iii) mild ovarian stimulation (mild-OS) cycle. Emerging evidence suggests that the optimal timing for FET using warmed blastocyst transfer is the LH surge+6 day, hCG administration+7 day, and the progesterone administration+6 day in the true natural cycle, modified natural cycle, and AC protocol, respectively. Although still controversial, better clinical pregnancy rates and live birth rates have been reported using the natural cycle (true natural cycle/modified natural cycle) compared with the AC protocol. Additionally, a higher early pregnancy loss rate and an increased incidence of gestational hypertension have been found in FETs using the AC protocol because of the lack of a corpus luteum. Although the common clinical practice is to employ luteal phase support (LPS) in natural cycles and mild-OS cycles for FET, the requirement for LPS in these protocols remains equivocal. Recent findings obtained from RCTs do not support the routine application of endometrial receptivity testing to optimize the timing of FET. More RCTs with rigorous methodology are needed to compare different protocols to prime the endometrium for FET, focusing not only on live birth rate, but also on maternal, obstetrical, and neonatal outcomes.

## 1 Introduction

Since its clinical application in 1978, in vitro fertilization (IVF) has been an efficient procedure of assisted reproductive technology (ART) that has provided a great opportunity for infertile couples worldwide to have children. Despite the development of optimal protocols for personalized, patient-specific stimulation and trigger as well as emerging technologies for improving embryo selection, the success rate for IVF in fresh transfer cycles remains low worldwide (1). With the technical improvement in cryopreservation using vitrification, frozen embryo transfer (FET) has become a viable alternative to fresh embryo transfer (2). The results obtained from laboratory-based studies indicate that the endometria of women in the controlled ovulation stimulation (COS) cycle are not appropriately prepared for embryo implantation (1). Several randomized controlled trials (RCTs) have shown that the clinical pregnancy rates (CPRs) following FET are slightly increased than those following fresh embryo transfer (3, 4). Additionally, elective cryopreservation in IVF cycles can prevent pregnancy-induced late ovarian hyperstimulation syndrome (OHSS) (5). For instance, an RCT study showed that in women with polycystic ovary syndrome (PCOS), FET had a higher live birth rate (LBR), a lower risk of OHSS, and a higher risk of preeclampsia than fresh transfer (6). Women with PCOS who underwent FET also had a lower early pregnancy loss rate compared to those who underwent fresh embryo transfer (6).

Furthermore, the perinatal and obstetric outcomes (including perinatal morbidity, small for gestational age, preterm birth, low birth weight, and antepartum hemorrhage) are less affected following FET (7). Compared to spontaneously conceived newborns, certain birth defects associated with abnormal blastogenesis were increased more than 3-fold in fresh embryo transfers but not in FET (8). However, it may take a longer time to achieve conception, given that embryo transfer is delayed in the FET cycle. Moreover, various obstetric outcomes, including maternal hypertensive disorders of pregnancy, having a large-for-gestational-age baby, and higher birth weight of the children born have been reported in women following FET implantation (1, 9).

During the past decade, cryopreservation of all embryos (or a “freeze-all” protocol) has become increasingly popular worldwide, given that it may overcome the detrimental effects of ovarian stimulation, especially in IVF patients who are at high risk of developing OHSS (5). Using cryoprotectants and rapid freezing, an optimized vitrification technique has become more widely applied for embryo cryopreservation, with a higher survival rate (up to approximately 95%) according to a large cohort study (10). The results obtained from meta-analyses showed that vitrified embryos had higher post-thawed survival rates (both for cleavage and blastocyst stages) than slow freezing embryos (11). Furthermore, clinical outcome comparison results showed that vitrification embryo transfers had higher CPR than slow freezing embryo transfers (12). The increasing number of IVF cycles to perform FET could partly reflect the upward trend in women receiving pre-implantation genetic testing cycles for aneuploidy detection (13, 14). Despite the increased trend in FET, the most optimal priming regimen of the endometrium in the general population during ART remains to be determined. To date, different endometrial preparation strategies have been proposed: i) natural cycle (NC) (true-NC with LH detection in blood or urine and modified-NC in which human chorionic gonadotropin (hCG) is administered to schedule embryo transfer instead of measuring LH); ii) artificial cycle (AC) or hormone replacement treatment using exogenous estradiol and progesterone; and iii) mild ovarian stimulation (mild-OS) cycle using gonadotropins, clomiphene citrate (CC), or letrozole.

With our systematic review, we aim to compare various endometrial preparation protocols for FET regarding reproductive, obstetric, and perinatal outcomes. In addition to the LBR, patient convenience and cost efficiency, pregnancy-related complications and perinatal health are also critical issues for infertile couples undergoing ART treatment.

## 2 Materials and methods

A comprehensive search was performed in the bibliographic databases PubMed and Embase, and the Cochrane Central Register of Controlled Trials (CENTRAL) from inception to May 31, 2023. Search terms included embryo cryopreservation, frozen embryo transfer, fresh embryo transfer, endometrial preparation, cryo-thawed, natural cycle, modified natural cycle, artificial frozen cycle, hormone replacement treatment, mild ovarian stimulation cycle, clinical outcome comparison, pregnancy outcomes, clinical pregnancy rate, live birth rate, early abortion rate, timing of FET, fresh ET after COS, delayed FET timing, and postpone FET timing. Full-text articles of relevant references were collected and assessed. The impacts of different protocols for FET on reproductive, obstetric, and perinatal outcomes are discussed based on the evidence derived from large prospective cohort studies RCTs, and meta-analyses. The literature search was restricted to articles published in English. The available articles were identified by manually searching the references of all relevance on this topic.

### 2.1 When is the optimal timing to perform FET after COS?

Given that there is a residual detrimental effect of COS on the receptivity of the endometrium, it has been suggested to postpone FET timing for at least a menstrual cycle following a successful fresh ET cycle or a free-all cycle (6, 15). Specifically, there is advanced endometrial maturation after COS leading to reduced implantation rates in fresh ET cycles compared to frozen or ET using donor oocytes in a RCT study (15). However, other researchers challenge the speculation that the residual impacts of COS on endometrial receptivity may persist until the next menstrual cycle. Indeed, delay of FET timing may induce emotional stress and anxiety in some patients, resulting in drop-out from their infertility treatment (16). A retrospective cohort study comparing two time points of FET showed that the implantation rates, CPRs, and LBRs were all reduced in the postponed FET compared to the immediate FET cycle (17). Similarly, the results obtained from a meta-analysis showed that the CPR and LBR were slightly higher in immediate FET than in postponed FET (18). A retrospective cohort study compared the immediate and delayed FET in high responder patients undergoing IVF cycles, and the results showed that there was no difference of pregnancy outcomes between two groups (19). Another retrospective study compared three time periods (immediate group < 40 days, delayed group >40 days but < 180 days, and overdue group > 180 days) of FET following freeze-all cycles, and the results showed that the time interval between oocyte retrieval and FET does not impact the pregnancy outcomes (20). However, these results were based on an analysis using retrospective cohort studies with the possible presence of selection bias. A RCT study compared the pregnancy outcomes of immediate FET and delayed FET among patients with a previous failed IVF/ET attempt, and the results showed that immediate FET had higher CPRs and LBRs than delayed FET (21). These findings suggest that unnecessarily delayed timing of FET should be avoid by shortening the time period of live birth during IVF treatment.

### 2.2 Natural cycle FET

Among the various FET protocols, the NC FET has gained popularity as a more physiological approach. The historical development and refinement of NC FET protocols have evolved over time to optimize success rates and improve patient outcomes (22). Although the concept of relying on a woman’s natural menstrual cycle for embryo transfer is not new, the advancements in assisted reproductive medicine have led to the standardization and optimization of NC FET protocols. Early attempts at NC FET were primarily based on spontaneous ovulation without any hormonal interventions, the true-NC FET. However, the lack of control over the timing of ovulation and the inability to accurately predict the optimal timing for embryo transfer limited the success of these early protocols. As a result, modifications were introduced to enhance the timing and precision of embryo transfer in NC FET, the modified-NC FET. Compared to the true-NC, the modified-NC is performed using hCG for ovulation triggering when the dominant follicle is reaching approximately 16-18 mm in diameter, which requires less precise hormonal and ultrasonographic monitoring.

### 2.3 True-NC FET

One significant advancement in the development of NC FET protocols was the introduction of ultrasound monitoring and hormonal assessments to track follicular development and predict the timing of natural ovulation (23). This allowed for improved timing of embryo thawing and transfer, ensuring synchronization between the embryo and the receptive endometrium. Over time, advancements in ultrasound technology and the development of more sensitive hormonal assays further refined the monitoring and assessment of the natural cycle (23). This improved the accuracy of predicting ovulation and provided a better understanding of the endometrial changes associated with receptivity. In practice, transvaginal ultrasonography is arranged on the cycle day 2 or day 3 to exclude any ovarian cyst or remaining corpus luteum from the previous cycle. Additionally, this baseline ultrasound helps assess the size of ovaries, the number of antral follicles, and the thickness of the endometrium. A baseline hormonal survey has been proposed to evaluate the patient condition and predict IVF outcomes (24), which has been challenged in later studies (23, 25, 26). A retrospective study observed that the acceptable baseline FSH level as a single test to predict IVF treatment failure is only obtained above a high-cutoff level (> 15 IU/L) (**Table 1**), indicating that the basal FSH level is of limited value in predicting IVF outcomes (26). It has been suggested that the treatment cycle should be canceled when the basal levels of estradiol (E2) and progesterone are elevated (E2 > 80 pg/ml or progesterone > 1.6 ng/ml) because of subsequently decreased pregnancy rates (27). However, this recommendation was based on the observation during the ovarian stimulating IVF cycle (27). The association studies regarding the impact of elevated basal E2/progesterone on the outcomes in NC FET are still pending.

**Table 1:** Ultrasonographic and laboratory features of monitoring ovulation in NC FET.

Ultrasonographic features of monitoring ovulation	
Target survey	Image finding
Dominant Follicle	Thinning and stretched appearance *
	Disappearance or sudden decrease in follicle size
	Corpus luteum formation
Douglas pouch	Release of fluid into the pelvic cavity
Endometrium	Homogenous or hyperechoic “luteinized endometrium”
*Impending rupture of the ovarian follicle	
Laboratory features of monitoring ovulation	
E2 (serum)	200-400 pg/ml (peaked 24 to 36 hours prior to ovulation)
LH (serum)	≥180% of last rising LH or ≥ 17 IU/l followed by a ≥ 30% drop in serum estradiol levels (peaked 10 to 12 hours prior to ovulation)
LH (urine)	> 20-40 IU/l (24 to 48 hours prior to ovulation)
Progesterone (serum)	1.5 ng/ml (confirmation of ovulation)

During true-NC preparation, serial ultrasounds are performed every other day (or daily) to monitor follicle size and growth starting on cycle day 8 (22). Transvaginal ultrasonography is a widely used standard method to monitor the development of ovarian follicles and predict ovulation time accurately. The dominant follicle, which is most likely to release the mature oocyte, is closely monitored. Typically, a follicle size of around 18 to 20 mm indicates that it is nearing maturity and ovulation is imminent. The exact size may vary based on individual factors and the specific protocols used by the fertility clinic. The thickness and appearance of the endometrium are also evaluated during transvaginal ultrasound. As ovulation approaches, the endometrium typically thickens (> 7 mm) and shows a triple-line pattern, indicating a good endometrial receptivity for embryo implantation (28). Under the observation using transvaginal ultrasonography, some key indicators can be used to predict or confirm ovulation: 1) the dominant follicle displays a thinning and stretched appearance, indicating impending rupture; 2) disappearance or sudden decrease in follicle size; 3) corpus luteum formation showing increased echogenicity inside the follicle; 4) the release of fluid into the pelvic cavity (Douglas pouch); and 5) Replacement of “triple-line pattern” of the endometrium by homogenous or hyperechoic “luteinized endometrium” (**Table 1**) (29, 30).

Serum hormonal levels can be utilized to predict ovulation by monitoring the levels of specific key hormones (LH, E2, and progesterone) that play a crucial role in the menstrual cycle. An observation study that included 23 normal endocrine women showed that serum E2 levels rapidly elevated during the late follicular phase, reaching a peak level (at approximately 200-400 pg/ml) 24-36 hours before ovulation (**Table 1**) (31). The peak level of E2 contributes to the positive feedback control leading to the pre-ovulatory LH surge in the female kisspeptin AVPV/PeN neuron (32). Almost at the time of the peak level of E2, the onset of the LH surge occurs (33). The LH surge triggers the final maturation and release of the oocyte from the follicle. Even in the same woman, variation in the ovulation timing exists from cycle to cycle. In this regard, the estimated timing of ovulation occurs at approximately 10 to 12 hours after the LH peak or 24 to 36 hours after the peak level of E2 (31). The onset of the LH surge seems to be the most reliable timing of impending ovulation, with a duration of 34 to 36 hours before follicle rupture (**Table 1**) (34). During the true-NC FET, monitoring LH levels through urine-based ovulation predictor kits (detection limits of 20-40 IU/l) or serum LH testing can help predict the timing of ovulation (**Table 1**). Adequate determination of the LH surge is critical to pinpoint the timing for FET. However, the standard criteria to define the LH surge is still pending without a consensus. The data obtained from a clinical study showed that any serum LH level equal to or exceeding 180% of the latest rising serum LH level is defined as a surge of LH (35). A retrospective study measuring serum levels of LH and estradiol demonstrated that the first detection of serum LH ≥ 17 IU/l followed by a ≥ 30% drop in serum estradiol levels (the next day after LH detection day) may indicate the timing of LH surge (**Table 1**) (36). Most commonly, a concomitant rise of serum P4 levels above 1.5 ng/ml detected on the day after the LH surge could be utilized to confirm ovulation (22). The appearance of LH detected in the urine is delayed in comparison with its detection in the peripheral blood (37). An observational study demonstrated that the mean time from peak serum LH to positive urine LH was 2 ± 2 hours, while the mean time from positive urine LH to follicular collapse was 20 ± 3 hours (38). Furthermore, the positive predictive values for ovulation within 24 or 48 hours following detecting urine LH were 73% and 92%, respectively (**Table 1**) (38). Examining serum progesterone levels is an effective method for predicting ovulation. Progesterone is a hormone produced by the corpus luteum, which forms after ovulation. A single serum progesterone level > 3 ng/ml in the mid-luteal phase has been used to retrospectively detect ovulation. Random serum progesterone of > 5 ng/ml has been proposed to confirm ovulation (sensitivity 89.6% and specificity 98.4%) (**Table 1**) (39).

Adequate secretion of progesterone by the corpus luteum formed from a dominant mature follicle is essential for preparing the receptive endometrium and maintaining a successful conception (40). The application of luteal phase support (LPS) in true-NC FET remained to be elucidated. The available retrospective studies revealed that some studies suggested the application of LPS (41), whereas other studies showed comparable reproductive outcomes in true-NC FET with or without LPS (42–44). A RCT demonstrated that administration of progesterone (vaginal micronized progesterone 400 mg bid) from the day (LH surge+3 day) of true-NC FET (cleavage-stage embryos) had a higher LBR than no LPS (45). Another RCT evaluated women undergoing cleavage-stage embryos using true-NC FET showed that hCG administration (one on the day of FET and one on the 6^th^ day of FET) for LPS did not increase the ongoing pregnancy rate compared to those without LPS (46).

Additionally, the use of exogenous hormonal agents, such as gonadotropin-releasing hormone (GnRH) agonists or antagonists, was incorporated into NC FET protocols to suppress premature ovulation and provide better control over the timing of ovulation. These medications helped to optimize the endometrial environment and enhance the chances of successful implantation. In recent years, there has been a growing interest in utilizing additional tools, such as endometrial biopsy or molecular markers, to further refine the assessment of endometrial receptivity in NC FET protocols (47, 48). These approaches aim to identify molecular markers or gene expression patterns associated with optimal endometrial receptivity, enhancing the selection of the most favorable timing for embryo transfer. Overall, the historical development of NC FET protocols has involved a gradual progression from spontaneous ovulation to refined approaches that incorporate ultrasound monitoring, hormonal control, and advanced assessment techniques. These advancements have improved the success rates of NC FET and have made it a viable option for patients undergoing assisted reproduction. Continued research and technological advancements are expected to further optimize NC FET protocols and contribute to its ongoing refinement.

In conclusion, the true-NC FET represents a promising alternative to traditional FET protocols, offering a more physiological approach with potential advantages in terms of patient experience, cost-effectiveness, and reduced treatment burden. While challenges and limitations exist, current evidence suggests that true-NC FET can achieve comparable success rates with carefully selected patients. Further research and refinement of the procedure are necessary to optimize patient selection, improve success rates, and ensure long-term safety. The true-NC FET holds great potential for the future of ART and warrants consideration as a valuable option in clinical practice.

### 2.4 Modified-NC FET

Modified-NC is an evolving technique that is considered more patient-friendly. Compared to the true-NC protocol, modified-NC is more flexible and requires less ultrasonographic and endocrine monitoring (**Table 2**). The initial monitoring in modified NC is similar to in true-NC; however, in a modified-NC, ovulation is triggered by the injection of hCG when the dominant follicle is between 16-20 mm in diameter. A RCT study including 60 IVF patients showed that the application of modified-NC significantly decreased the number of clinical visits (for cycle monitoring) without affecting the reproductive outcomes (47). To date, there is no consensus regarding the dosage (from 5000 IU to 10000 IU) used to trigger ovulation in the modified-NC protocol. A RCT four-arm study compared three different dosages of hCG (5000, 6500, and 10000 IU) to trigger ovulation during the GnRH antagonist short protocol IVF treatment (49). The results showed that increasing hCG trigger doses (6500-10000 IU) significantly increased endogenous progesterone concentration during the mid- to late-luteal phase (49). These findings suggest that the administration of hCG may act as an ovulation trigger and also as a promotor of luteal phase support. A retrospective cohort study comparing true-NC and modified-NC cycles revealed that modified-NC displayed significantly higher implantation rate, CPR, and LBR (50). However, a RCT contradicted this finding and was terminated early because the results obtained from an interim analysis showed a significantly higher ongoing pregnancy rate in the true-NC group than in the modified-NC group (51).

**Table 2:** Characteristics of different FET protocols.

	True-NC	Modified-NC	Mild-OS	AC
**Endometrial preparation**	■ Day 2/3 ultrasonography to exclude ovarian cyst/corpus luteum ■ Day 2/3: consider cancelation if FSH>15 IU/L ■ Day 8: monitor follicular growth daily or every other day until features of ovulation	■ Day 2/3 ultrasonography to exclude ovarian cyst/corpus luteum ■ Day 2/3: consider cancelation if FSH>15 IU/L ■ Day 8: monitor follicular growth daily or every other day until dominant follicle 16-20 mm; and triple-line pattern	■ Day 2/3: CC/letrozole or/and gonadotropin ■ Day 8: monitor follicular growth daily or every other day until dominant follicle 17-18 mm and EM >7 mm	■ Day 1/2/3: estradiol step-up/fixed-dose regimen ■ Day 8: monitor endometrial receptivity by ultrasonography
**Timing of embryo transfer**	■ Confirmation of ovulation by ultrasonography and laboratory findings ■ Blastocyst transfer -LH surge Day+6 -Ovulation Day+5	■ Ovulation triggered by hCG ■ Blastocyst transfer -hCG trigger Day+7	■ Ovulation triggered by hCG ■ Blastocyst transfer -hCG trigger Day+7 ■ Day 3 embryo -hCG trigger Day+5	■ Ultrasonography monitoring for FET -EM >7 mm -EM “triple line” pattern -EM good blood flow ■ Blastocyst transfer -Progesterone administration Day+6
**Luteal support**	■ Might have some benefits, but more studies needed	■ Might not have benefits, but more studies needed	■ Might have some benefits, but more studies needed	■ Continue progesterone administration until luteo-placental shift
**Advantages**	■ More physiological approach ■ Cost-effectiveness ■ Reduced treatment burden	■ More flexible ■ Require less ultrasonographic and endocrine monitoring	■ More favorable endocrine environment ■ Improved endometrial receptivity	■ More convenient and flexible to schedule FET ■ A lower cycle cancellation rate

Similar to the situation in true-NC, there is a debate regarding whether LPS is needed in modified-NC. Given its long half-life up to at least 7 days, hCG administration may have an adequate luteotropic effect during the early luteal phase (52). Theoretically, the application of LPS is not required in the modified-NC FET, given that too early progesterone supplementation may cause asynchrony between the embryo and endometrium, leading to adverse reproductive outcomes (42, 53, 54). In this regard, two retrospective studies did not show any improved reproductive outcomes with the application of LPS in modified-NC (55, 56). In line with these results, two RCTs also demonstrated no beneficial effects of LPS on the reproductive outcomes following modified-NC FET (57, 58). Emerging evidence suggests that when LPS is applied in modified-NC, the administration of progesterone should not be started earlier than the LH surge+3 day (42, 45).

### 2.5 What is the optimal timing for FET in true-NC and modified-NC protocols?

The optimal window of embryo implantation may vary from day 16 to day 20 for cleavage-stage (2-12 cells) embryos in a 28-day cycle (59–62). The thawed embryos may take a longer time to develop into the blastocyst stage because these embryos lose approximately more than half the cell viability of their blastomeres (63). Therefore, the window of embryo transfer in a NC ranges from LH+7 to LH+11 (63). In this regard, there is a time difference in FET between true-NC and modified-NC, given that ovulation occurs 24 to 36 hours after a spontaneous LH surge, while 36 to 48 hours after hCG administration (64). In clinical practice, FET using blastocyst-stage embryos is arranged on ultrasonographic confirmation of ovulation+5 days or LH surge+6 days in the true-NC and hCG administration +7 days in the modified-NC, respectively (22). A retrospective study measured the spontaneous LH surge (≥ 20 mIU/ml) to determine the timing of modified-NC, suggesting that FET was scheduled on hCG+6 days with a documented LH surge, whereas FET was scheduled on hCG+7 days without an LH surge (65). Similarly, a multicenter-RCT evaluated the optimal timing for modified-NC FET indicated that embryo transfer should be arranged on LH+6 days in true-NC and hCG+7 days in modified-NC (**Table 2**) (65).

In summary, based on the literature review, FET can be scheduled on ovulation+5 day or LH+6 day in true-NC protocol and hCG+7 day in modified-NC protocol using blastocyst-stage embryos (**Table 2**). For day 3 cleavage-stage embryo transfer, FET can be scheduled on ovulation+3 day or LH+4 day in true-NC protocol and hCG+5 day in modified-NC protocol.

### 2.6 Mild ovarian stimulation cycle

Mild-OS cycle for FET aims to reduce the supraphysiological levels of hormones observed in high-dosage protocols and improve subtle defects in folliculogenesis, leading to a more favorable endocrine environment and improved endometrial receptivity (**Table 2**) (66, 67). Appropriate patient selection is crucial for the successful implementation of mild-OS in FET cycles. Factors such as age, ovarian reserve, previous response to ovarian stimulation, and the number and quality of cryopreserved embryos should be considered when determining the suitability of a patient for mild-OS (67). Tailoring mild-OS protocols to individual patient characteristics is essential to optimize outcomes. In mild-OS FET, either oral ovulatory agents (clomiphene citrate or CC and letrozole), exogenous gonadotropins, or in combination, can be used to prepare the endometrium for embryo transfer. Several ovulation induction protocols have been proposed in the mild-OS cycle, which includes CC at a dosage of 50-100 mg per day, letrozole at a dosage of 2.5-5 mg per day, and recombinant/urinary FSH at a low dosage of less than 150 IU per day, starting on the second or third day of the menstruation (22). Letrozole is an aromatase inhibitor that was initially introduced as an alternative ovulation induction agent in the early 2000s. Unlike CC, letrozole has the advantage of without interference with endometrial development and has been reported to be more effective in obese women with PCOS (68). Similar to CC, not all patients respond to a 5-day regimen of letrozole administration. Therefore, extended regimens (7–10 days), stair-step protocols, and in combination with low-dosage recombinant gonadotropins have been proposed (69).

Close monitoring and adjustment of medication dosages based on ovarian response using vaginal ultrasonography and hormonal profiles are then performed after medication. Similar to modified-NC, hCG is administrated when the leading follicle reaches 17-18 mm in diameter, the serum E2 level is more than 150 pg/ml, or the endometrial thickness is more than 7 mm (22). Eventually, FET can be scheduled on hCG+7 day for blastocyst-stage embryos or hCG+5 day for day-3 embryos (**Table 2**). Although most clinicians are applying LPS in mild-OS cycle FET (70), more well-designed RCTs are needed to evaluate the place of LPS in the mild-OS cycle for FET.

### 2.7 Artificial cycle or hormone replacement treatment

AC or hormone replacement treatment for FET involves the administration of exogenous estrogen and progesterone to stimulate the growth of the endometrium and inhibit follicular growth. Initially, estrogen is given to stimulate endometrial growth, followed by progesterone administration to prepare the endometrium for embryo transfer. Compared to other protocols, the AC protocol is more convenient and flexible for both patients and physicians to schedule embryo transfers with a lower cycle cancellation rate (**Table 2**) (71). However, the disadvantages AC FET are some potential detrimental effects induced by the supplementation of exogenous estrogen and the absence of the corpus luteum.

2.8 Administration of estrogen

In AC, estrogen administration is usually started within the first three days of a menstrual cycle to prime the endometrium. Estradiol can be given as a fixed-dose or in a step-up regimen. The fixed-dose regimen is utilized to prevent follicular growth and ovulation, whereas the step-up regimens can increase estradiol exposure in a more physiologic manner (71). The fixed-dose regimen is given at a dosage of 6 mg per day starting on the first, second, or third day of the cycle. The dosage of estradiol in the step-up regimen varies, but is most commonly given starting at 2 mg per day during the first week, then increased to 4 mg per day for the following 5 days, and finally step-up to 6 mg per day until the day of embryo transfer (72). A large-scale retrospective study included 8254 cycles and compared two regimens (fixed-dose and step-up regimens) in oocyte donation cycles using both oral and transdermal routes (72). The results showed that there was no significant difference between the two groups in terms of LBR (oral: 33% vs 32.5%; transdermal: 35.7% vs 32%, respectively) (72). A more recent retrospective study included 394 cycles and compared three regimens used for estradiol administration: one fixed-dose regimen using 6 mg per day and two step-up regimens (one regimen received 2 mg per day for 6 to 7 days, then 4 mg per day for 4 to 5 days, and 6 mg per day until ET; the other regimen received 4 mg per day for 7 to 8 days, then 6 mg per day until ET) (73). They found that the step-up regimen starting with 4 mg per day resulted in the greatest endometrial thickness, compared to the step-up regimen using 2 mg per day and the fixed-dose regimen using 6 mg per day (10.2 ± 1.3 mm vs. 9.6 ± 1.4 mm vs. 8.6 ± 0.9 mm; P < 0.001) (73). Furthermore, the reproductive outcomes also favored the 4 mg step-up regimen, with the highest CPR (55.2% vs. 41.1% vs. 42.2%; P <0.035) and highest LBR (50.9% vs. 40.8% vs. 48.1%; P=0.320) (73). There seems to be a trend favoring the 4 mg step-up regimen over the 2 mg step-up regimen or the fixed-dose regimen in terms of endometrial thickness and reproductive outcomes.

Different routes of estrogen administration (oral, transdermal, and vaginal) have been proposed to employ in AC FET based on individual patient characteristics, with the oral form being the most widely used route (74). The Cochrane Database of Systematic Reviews compared the equivalent of 6 mg estradiol daily in oral and two different transdermal forms and found comparable reproductive outcomes among these different administration routes (75). A prospective study compared the efficacy of transdermal estrogen (gel) with oral estradiol in AC FET and found that there was no difference in endometrial thickness, implantation rates, CPR, and miscarriage rate; however, the transdermal form had a better patient satisfaction rate (8.02 ± 1.07 vs 6.96 ± 0.99, p<0.01), and the related side effects were significantly lower (18.1% vs 55.1%, p<0.01) (76). A retrospective cohort study compared the effects on the endometrial preparation using oral and vaginal tablets of estrogen in AC FET, and they found a thinner endometrium on the day of transfer in the vaginal form group, but the two groups had similar reproductive outcomes (77). A small, randomized study compared the effects of oral and vaginal estrogen administration on endometrial preparation [evaluating endometrial histology by hematoxylin and eosin staining and immunohistochemical analysis of estrogen receptor (ER) expression] in patients with primary ovarian insufficiency (78). The results showed that patients using vaginal estrogen for 14 days had higher E2 concentrations, thicker endometrial thickness, and more pronounced ER expression compared to those who used oral estrogen (78). However, oral estrogen remains the most popular application because of its convenience. Future well-designed studies comparing the effects of different routes of estrogen administration on endometrial proliferation, adverse impacts, and risk of thrombosis will be of great interest.

The range of the duration of estrogen priming varies greatly, from 6 days to 36 days, providing flexibility to extend the length of the follicular phase to achieve adequate endometrium for implantation (79). A retrospective study analyzed 4142 FET cycles and compared the 7-day and 14-day estrogen administration, and the results showed both regimens achieved adequate endometrial preparation with comparable reproductive outcomes (80). However, there might be a negative impact on reproductive outcomes if the duration of estrogen priming is too short. A retrospective study included 835 oocyte donation cycles, which were divided into five groups by 10-day increments according to the length of estrogen priming before implantation (81). The results showed that the implantation rates and pregnancy rates among the five groups were similar (81). However, the duration of estrogen administration that was less than 10 days had a significantly higher miscarriage rate (41%, p=0.04) compared to the other four groups of patients who received estrogen administration for longer duration (81).

### 2.9 Administration of progesterone

In AC for FET, the optimal timing for progesterone administration is critical to ensure proper endometrial preparation and synchronization with the embryo transfer. The timing varies depending on the specific AC protocol and the preferences of the treating physician. Using various techniques (ultrasonography, hysteroscopy, histology, immunobiological staining, and endometrial receptivity array), several markers of endometrial receptivity have been proposed to evaluate the implantation window (28). Among these tools, transvaginal ultrasonography is the most popular and non-invasive technique to evaluate endometrial receptivity in AC protocol. Associations have been identified between clinical pregnancy and various endometrial receptivity markers including endometrial thickness, endometrial pattern, Doppler indices, endometrial wave-like activity, and various molecules; however, their poor ability to predict clinical pregnancy prevents them from being used as diagnostic tests of endometrial receptivity (28). In AC FET, the endometrial thickness (> 7 mm), endometrial pattern (the triple line pattern), and endometrial blood flow (presence of endometrial blood flow) are the most common markers for good endometrial receptivity evaluated by transvaginal ultrasonography (**Table 2**) (28). When these markers have been detected, progesterone supplementation is commenced, and the timing of FET is scheduled accordingly.

In patients undergoing AC FET, optimal exposure to progesterone supplementation is critical for a successful conception. However, insufficient data were available to apply the route, dosage, and starting date of progesterone during the AC FET cycle. Progesterone can be administered through various routes, including oral, rectal, intramuscular, subcutaneous, and vaginal (suppositories or gels). The specific route of administration is determined based on individual patient characteristics and clinic preferences (82–88). Each route has its advantages and considerations, such as convenience, patient comfort, and absorption rates. Additionally, various available forms can be used to support endometrial receptivity, including micronized capsules, tablets, suppositories, and bio-adhesive gels. To date, there is still insufficient comparative data regarding the effects of different routes or dosages of progesterone supplementation on the subsequent reproductive outcomes. Among different routes of progesterone, vaginal administration is the most used route. A retrospective study including 346 patients undergoing AC FET cycles compared two regimens of bio-adhesive progesterone gel (90 mg per day and 180 mg per day) and found that the implantation rates and LBRs were significantly higher in the 180 mg per day group (88). Similarly, a retrospective study including 2100 AC FET cycles compared different dosages (900 mg per day and 1200 mg per day) of oral progesterone capsules and found that patients using the dosage of 1200 mg per day had higher CPR (89). These findings suggest that adequate progesterone supplementation during AC FET can reduce the incidence of early pregnancy loss, leading to a significantly higher LBR. In terms of reproductive outcomes, there is a lack of consensus regarding which progesterone administration route is more efficient. Two RCTs have compared intramuscular and vaginal routes in patients undergoing AC FET and found similar CPRs (90, 91). Some retrospective studies observed improved reproductive outcomes in patients using the intramuscular route than those using the vaginal route (92, 93). However, other retrospective studies reported similar reproductive outcomes comparing intramuscular and vaginal routes (94, 95). Interestingly, a RCT compared three different regimens (50 mg per day intramuscular progesterone only; 400 mg per day vaginal progesterone only; and 400 mg per day vaginal progesterone plus 50 mg intramuscular progesterone every 3rd day) in AC FET cycles using vitrified blastocyst transfer (96). The results obtained from the interim analysis showed that patients receiving vaginal progesterone only had a lower CPR (31% vs. 50% vs. 47%) and LBR (27% vs. 44% vs. 46%) compared to the other two groups (96, 97). Another retrospective study including 1364 AC FET cycles compared reproductive outcomes of two regimens (400 mg twice per day vaginal micronized progesterone plus 10 mg twice per day oral progesterone and 400 mg twice per day vaginal micronized progesterone only) (98). Significantly lower abortion rate (3.4% vs. 6.6%) and higher LBR (46.3% vs. 41.3%) were noted in the group of combined vaginal and oral routes progesterone (98). Without a doubt, more RCTs are needed to clarify the best regimen and optimal dosage of various progesterone supplementation for AC FET.

The supplementation of progesterone for luteal support is particularly crucial in AC FET cycles because of the lack of endogenous progesterone secreted by the corpus luteum. There is a debate regarding the optimal duration of luteal support applied in AC FET cycles after conception (99, 100). Theoretically, progesterone supplementation should be continued until the time of luteo-placental shift (at approximately 10-12 weeks of gestation) when the placental tissue is able to produce enough endogenous progesterone (**Table 2**) (100).

### 2.10 Administration of GnRH analogs

In AC FET, GnRH analogs have been proposed to control the hypothalamus-pituitary-ovary axis and optimize the endometrial environment. GnRH analogs, such as GnRH agonists and GnRH antagonists, play a crucial role in suppressing the endogenous hormonal fluctuations and preventing premature ovulation (101). GnRH agonists initially cause a temporary surge in gonadotropin release before desensitizing the pituitary gland. Continuous administration of GnRH agonists results in a decrease in the reaction of GnRH because of an obvious uncoupling of GnRH receptors followed by the downregulation of GnRH receptors (102). Therefore, prolonged exposure to GnRH agonists desensitizes the GnRH receptors, leading to decreased FSH and LH secretion from the pituitary gland. This suppression of gonadotropin secretion prevents the development of the dominant follicle and subsequent ovulation (101). GnRH antagonists competitively bind to and block GnRH receptors on the gonadotroph cells of the pituitary gland. This binding prevents the endogenous GnRH from activating the receptors and inhibits the downstream signaling pathways that lead to the release of FSH and LH (103). A single-center RCT included 473 FET cycles and compared the 7-day dosage of GnRH antagonist with a single dose of long-acting GnRH agonist (104). The authors found that there was no significant difference in ongoing pregnancy rate between the two regimens and concluded that the outcomes are similar between GnRH antagonist and down-regulated hormone replacement protocols for women with ovulatory cycles undergoing oocyte donation (104). Although the addition of GnRH analogs for ovulation suppression is highly efficient, the AC FET protocol without GnRH analogs-induced suppression is more popular because of patient friendly. However, compared to GnRH analog application, AC FET without GnRH analogs has been reported with an incidence of approximately 1.9% to 7.4% of cycle cancellation because of the occurrence of premature ovulation (105, 106). A RCT included 234 patients undergoing FET cycles that compared AC protocols with or without GnRH agonist suppression (107). The results showed that AC with GnRH suppression had a higher LBR per initiated cycle than AC without GnRH suppression (107). However, the results obtained from the Cochrane Review database showed that there was no significant difference between the two protocols (AC with GnRH suppression and AC without GnRH suppression) in terms of CPRs, cycle cancellation rates, miscarriage rates, and endometrial thickness (75). A retrospective cohort study included 9263 women who underwent FET with or without long-acting GnRH agonist administration before AC protocol and compared the pregnancy outcomes (108). The results showed that for those who had no or one failure of embryo implantation, there was no difference in LBR between AC with GnRH agonist pretreatment and AC without GnRH agonist pretreatment (108). However, for those who had multiple failure of embryo implantation, AC protocol with GnRH agonist pretreatment resulted in a higher LBR than AC protocol without GnRH agonist pretreatment (108).

### 2.11 Reproductive outcomes after different FET protocols

#### 2.11.1 Reproductive outcome comparison between NC (true-NC/modified-NC) and AC FET

**Figure 1:**
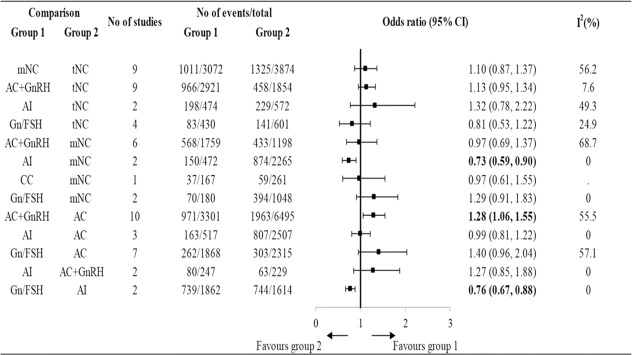
The reproductive outcomes of live birth rate among different endometrial preparation protocols in cohort studies. t-NC, true natural cycle; m-NC, modified natural cycle; AC, artificial cycle without suppression; AC+GnRH, artificial cycle with gonadotropin-releasing hormone cycle; Gn/FSH, ovarian stimulation with gonadotropin or follicle stimulating hormone; AI, aromatase inhibitor; CC, clomiphene citrate [Reproduced with permission from SPRINGER/PLENUM PUBLISHERS, Reference (122)].

Based on the results analyzed from available cohort studies (retrospective or prospective), most studies reported similar reproductive outcomes between NC (true-NC and modified-NC) FET compared with AC FET (109–113). However, there were some controversial results given that some studies reported better outcomes (114–116) or worse outcomes (117, 118) in patients receiving true-NC/modified-NC than those receiving AC FET. Among the abovementioned protocols, studies have shown that AC FET resulted in the highest early pregnancy loss (109, 119). A retrospective cohort study included 634 FET cycles and compared the early pregnancy loss incidence in patients using AC FET or true-NC FET (120). The results showed that AC FET protocol had a higher early pregnancy loss rate than true-NC FET (54.7% versus 33%, P<0.0001) (120). Similarly, a meta-analysis comparing the obstetric outcomes after NC versus AC FET showed that patients using AC FET protocol had a higher early pregnancy rate (121). A total of 26 RCTs and 113 cohort studies were included in a network meta-analysis, which compared 7 different FET protocols: true- NC; modified-NC; AC with GnRH agonist suppression; AC without GnRH agonist suppression; aromatase inhibitor (letrozole); clomiphene citrate; and exogenous gonadotropin (122) (**Figure 1**). Using a network meta-analysis, the results showed that AC ranked as the lowest LBR compared with the other protocols (122) (**Figure 1**). Using a pairwise meta-analysis of observational studies, AC was associated with significantly lower LBRs compared with true-NC and modified-NC (122) (**Figure 1**). However, the results obtained from a Cochrane review meta-analysis including 5 RCTs revealed a trend, yet not reaching statistic difference, showing a higher CPR in AC than NC (75). Of note, a RCT included 959 FET cycles showed that patients receiving AC FET had a higher cancellation rate than those receiving modified-NC (124/464 versus 101/495, OR 1.4, 95% CI 1.1–1.9, *P* = 0.02) (123). In this regard, the increased cancellation rate found in the AC FET protocol was due to insufficient endometrial thickness. However, the analytic data showed a similar cost after receiving AC or modified-NC (123). In summary, dada obtained from available studies indicate that the reproductive outcomes in NC (true-NC and modified NC) were slightly better than those in AC with low-quality evidence.

#### 2.11.2 Reproductive outcome comparison between mild-OS and AC FET

Using transvaginal ultrasonography to measure the endometrial thickness is the most common clinical approach to evaluate endometrial receptivity (28). A retrospective study compared two regimens of endometrial preparation in 2664 women with PCOS undergoing FET (124). The results showed that the endometrial thickness on the day of progesterone supplementation and on the day of embryo transfer was significantly thicker in patients receiving mild-OS with letrozole than in those receiving AC (124). Additionally, after adjusting the related confounding factors, this study demonstrated that LBR was significantly higher, and the early pregnancy loss rate was lower in the letrozole group compared with the AC group (124). In contrast to these results, the other two studies did not show any significant difference in endometrial thickness when letrozole FET was compared with AC FET (125, 126). The endometrial thickness was significantly thinner in mild-OS with CC than in AC (75). Using a pairwise meta-analysis, a Cochrane database showed that the CPR was significantly higher in the mild-OS (letrozole or clomiphene citrate) FET when compared to AC FET (75). However, no significant difference was found in CPR between the mild-OS with clomiphene citrate and AC FET (75). Further meta-analysis showed that the CPR was significantly higher in the mild-OS with letrozole FET when compared to AC FET (75). The reproductive outcome focusing on LBR has been compared between two regimens, mild-OS with letrozole and AC, in an available RCT with a limited sample size (127). The results showed a similar LBR in both mild-OS with letrozole and AC, with low-quality evidence (127). A network meta-analysis including 26 RCTs compared 7 different regimens of FET, and the results showed that significantly higher LBRs were observed in mild-OS with letrozole or mild-OS with gonadotropin compared to the AC (122). More RCTs are required to support the feasible application of mild-OS in patients undergoing FET.

#### 2.11.3 Obstetric and neonatal outcome comparison between NC (true-NC/modified-NC) and AC FET

A most recent meta-analysis included 30 RCTs and cohort studies, which compared the obstetric and neonatal outcomes following NC FET and AC FET (121). The results obtained from the meta-analyses showed that the average neonatal birthweight was lower following NC FET compared to AC FET (121). Compared to AC FET, newborns delivered from NC FET cycles had a lower risk of various neonatal outcomes, including large for gestational age, low birth weight, and macrosomia (121). Furthermore, pregnant women following NC FET had a lower risk of multiple obstetric outcomes, including early pregnancy loss, preterm birth, very preterm birth, pregnancy-induced hypertension, pre-eclampsia, placenta previa, and postpartum hemorrhage (121). Using a pairwise comparison analysis, a network meta-analysis (including 26 RCTs and 113 cohort studies) that compared different FET protocols showed that infertile patients who achieved pregnancy using AC had an increased risk of developing pregnancy-induced hypertension, postpartum hemorrhage, and preterm labor, compared with those using true-NC (122) (**Figure 1**). Intriguingly, data obtained from stratified analyses showed that NC FET with LPS significantly decreased the risk of preterm labor (but not other obstetric outcomes) whereas, NC FET without LPS did not decrease this risk (121). Because of the very low quality of evidence, the efficacy of the use of LPS in NC FET remains to be elucidated by using a large-scale RCT.

#### 2.11.4 The application of endometrial receptivity testing in FET cycles

Endometrial receptivity refers to the state of the endometrium that is conducive to embryo implantation and is a complex process involving molecular, cellular, and structural changes in the endometrium (128). The receptive window, known as the window of implantation (WOI), is a limited timeframe during which the endometrium is receptive to embryo attachment and subsequent implantation (129). Deviations from the optimal receptivity window can lead to implantation failure and reduced pregnancy rates. Through the endometrial transcriptomic analyses, researchers have identified several differentially expressed genes among all phases of the menstrual cycle, including the receptive phase (130, 131). In this regard, endometrial receptivity testing is a diagnostic technique that uses transcriptomic analyses to classify the endometrial biopsy samples into pre-receptive, early receptive, receptive, late receptive, or post-receptive (47). The objective of this technique is to determine the optimal for conducting a FET for an individual patient, considering when progesterone exposure begins (47). To date, hundreds of thousands cycles of endometrial receptivity testing have been carried out; however, the results on reproductive outcomes have yielded inconsistent results. Some studies indicate that receptivity-timed FET may lead to better outcomes compared to standardized timing, while others have found no significant difference (47, 132–134). A RCT that included 767 AC FET cycles using at least one euploid blastocyst was conducted to evaluate whether timed FET according to endometrial receptivity testing improves reproductive outcomes relative to standardized FET (135). The primary outcome, LBR, was observed in 58.5% of transfers (223 out of 381) in the intervention group, compared to 61.9% of transfers (239 out of 386) in the control group, with no significant difference between the two groups (p=0.38) (135). No significant differences were found between the intervention and control groups for the predetermined secondary outcomes, including the rate of biochemical pregnancy (77.2% vs 79.5%, p=0.48) and CPR (68.8% vs 72.8%, p=0.25) (135). Taken together, the utilization of endometrial receptivity testing to determine the optimal timing of FET did not result in a significant improvement in the LBR among patients with euploid blastocysts from IVF. These findings do not provide substantial evidence to support the routine use of receptivity testing for guiding the timing of embryo transfer in the context of IVF/ET.

## 3 Conclusion

Despite the global rise in FET for various indications, there is an ongoing quest to determine the optimal protocol for preparing the endometrium. While NC (true-NC/modified-NC) and AC FET are the commonly utilized protocols, it is crucial to conduct well-designed and powerful RCTs comparing different protocols to optimize endometrial preparation for FET. These trials should not only focus on LBR or CPR but also assess maternal, obstetrical, and neonatal outcomes. Currently, limited-quality evidence indicates that the NC (t-NC/modified-NC) may be superior to AC. Furthermore, caution is advised with AC due to a potential incidence of early pregnancy loss reported in some studies, and recent evidence indicates an increased risk of developing hypertensive disorders in pregnancies because a lack of the corpus luteum. More RCTs are needed to clarify the best regimen and optimal dosage of various estrogen and progesterone supplementation for AC FET and the application of LPS for NC. Regarding the timing of warmed blastocyst transfer, evidence suggests that the optimal timing for FET can be LH surge+6 day, hCG administration+7 day, and the progesterone administration+6 day in the true-NC, modified-NC, and AC protocols, respectively (**Table 2**). It is important to further explore time adjustments considering individual variations in the WOI or the day of vitrification. Finally, emerging evidence does not support the routine application of endometrial receptivity testing for guiding the timing of FET in different protocols.

## Author contributions

Y-WH: Writing – original draft, writing – review, visualization.C-CH: Writing – original draft, Visualization. S-WH: Writing – review, visualization. C-WC: Writing – original draft, visualization. H-CH: Writing – original draft, visualization. T-CY: Conceptualization, writing – review and editing, visualization. W-CL: Conceptualization, writing – review and editing, visualization. S-YS: Supervision, writing – review and editing, visualization. H-MC: Conceptualization, supervision, writing – review and editing, visualization. All authors contributed to the article and approved the submitted version.
